# Characterization of intrinsically disordered regions through scalar coupling-based solid-state NMR experiments

**DOI:** 10.52601/bpr.2025.240065

**Published:** 2025-08-31

**Authors:** Tong Zeng, Juan Li, Chaowei Shi, Shengqi Xiang

**Affiliations:** 1 MOE Key Lab for Cellular Dynamics, School of Life Sciences, Division of Life Sciences and Medicine, University of Science and Technology of China, Hefei 230022, China; 2 Hefei National Laboratory for Physical Sciences at Microscale, University of Science and Technology of China, Hefei 230022, China

**Keywords:** Solid-state NMR, IDR, INEPT, Amyloid fibrils, Scalar coupling

## Abstract

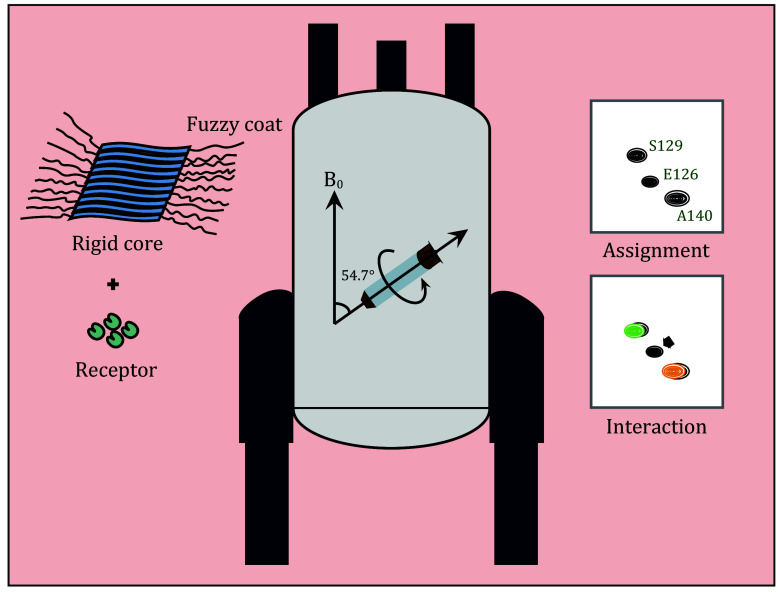

Abnormal amyloid fibrils are characteristic features and common pathological mechanisms of various neurodegenerative diseases, often found in disease-related brain regions, leading to neuroinflammation and neuronal apoptosis. Many disease-associated amyloid fibrils consist of a rigid fibril core primarily composed of cross-β sheets, surrounded by a fuzzy coat formed by intrinsically disordered regions (IDR). Over the past two decades, substantial structural knowledge of the rigid fibril core has been accumulated through cryo-electron microscopy (cryo-EM) and solid-state nuclear magnetic resonance (ssNMR) based on cross-polarization. In contrast, the highly disordered conformations of the fuzzy coats have hindered their structural characterization. Here, we describe the application of two-dimensional (2D) heteronuclear single quantum coherence (HSQC) and three-dimensional (3D) HNCO, HNCA, and HN(CO)CA spectra, utilizing the scalar coupling-based ^1^H detection magic angle spinning (MAS) ssNMR techniques for backbone assignment of the IDR in amyloid fibrils, with the aim of further elucidating the conformational changes of the IDR during ligand binding processes.

## INTRODUCTION

An increasing body of research indicates that the pathogenic mechanisms of various neurodegenerative diseases involve the misfolding, aggregation, and deposition of proteins (Eisenberg and Jucker 2012; Soto 2003). For instance, the brains of patients with Alzheimer's disease (AD) contain amyloid-β (Aβ)-derived neuritic amyloid plaques and hyperphosphorylated tau protein aggregates known as neurofibrillary tangles (Lu *et al.* 2020; Stancu *et al.* 2019). In patients with Parkinson's disease (PD), aggregates of Lewy bodies, primarily composed of α-Synuclein (α-Syn), are found in the cytoplasm of neurons in the substantia nigra (Luk *et al.* 2012; Zhao *et al.* 2020a). Abnormal accumulations of TAR DNA-binding protein 43kDa (TDP-43) are present in the neurons and glia of patients with amyotrophic lateral sclerosis (ALS) and various forms of frontotemporal lobar degeneration (FTLD) (Arseni *et al.* 2021). Additionally, deposits of huntingtin protein can be observed in the nuclei of brain regions affected by Huntington's disease (HD) (Soto 2003). Although these proteins exhibit no homology in their sequences or structures, they all manifest as amyloid fibrillar networks in brain regions associated with degenerative diseases, further disrupting protein quality control systems and triggering neuroinflammation and neuronal apoptosis (Scheres *et al.* 2023).

Abnormal amyloid fibrils serve as critical pathological entities, and their high-resolution structures are essential for understanding their formation, assembly, and underlying pathological mechanisms. Over the past two decades, the structures of various amyloid fibrils have been extensively studied using cryo-electron microscopy (cryo-EM) (Gremer *et al.* 2017; Li *et al.* 2018; Long *et al.* 2021; Sun *et al.* 2020; Zhao *et al.* 2020b). For example, Fitzpatrick and colleagues constructed atomic models of helical and straight tau core filaments derived from the brains of AD patients based on cryo-EM images with a resolution of 3.4–3.5 Å (Fitzpatrick *et al.* 2017). Schweighauser and colleagues utilized cryo-EM to elucidate two types of α-Syn fibrils from the brains of patients with multiple system atrophy (MSA), discovering that the conformations of these fibrils differ from those found in dementia with Lewy bodies (DLB), suggesting that distinct conformations may characterize specific synucleinopathies (Schweighauser *et al.* 2020). Arseni and colleagues reported the cryo-EM structure of aggregated TDP-43 in the brains of ALS and FTLD patients (Arseni *et al.* 2021). Yang and colleagues employed cryo-EM to determine the fibrillar structures of two types of Aβ42 derived from human brains (Yang *et al.* 2022). The elucidation of these amyloid fibril structures aids the development of diagnostic and therapeutic agents for related diseases.

Amyloid fibrils are insoluble, making them unsuitable for analysis via solution nuclear magnetic resonance (NMR) (Sanders and Sönnichsen 2006; Tamm and Liang 2006). Solid-state NMR (ssNMR) serves as a valuable technique for investigating the structure of amyloid fibrils (Helmus *et al.* 2010; Naito and Kawamura 2007; Parthasarathy *et al.* 2011; Wickramasinghe *et al.* 2021). The ssNMR experiments enhance the signal intensity of nuclei with low gyromagnetic ratios, such as ^13^C and ^15^N, within amyloid fibrils through cross-polarization (CP). The samples are subjected to magic angle spinning (MAS) at a 54.7° angle to mitigate spectral line broadening caused by various anisotropic effects (Stejskal *et al.* 1977). Furthermore, the ssNMR experiments effectively eliminate dipolar interactions between ^1^H and X nuclei through the application of high-power proton decoupling methods. The combination of CP, MAS, and high-power decoupling enabled the high-resolution spectra of amyloid fibrils (Knight *et al.* 2011; Zhou *et al.* 2012). Wasmer and coworkers utilized ssNMR to constrain structure from 134 intramolecular and intermolecular experimental distances, revealing that the HET-s protein (residues 218 to 289) forms a left-handed β-helical amyloid fibril structure (Wasmer *et al.* 2008). Lu and coworkers conducted ssNMR studies on Aβ fibrils amplified from the seed extracted from the brains of two Alzheimer's disease (AD) patients, demonstrating that the molecular structures of the two Aβ fibrils differ, which may correlate with changes associated with AD (Lu *et al.* 2013). Heise and coworkers assigned the 48 residues in the hydrophobic core region rich in β-sheets within α-Syn fibrils through MAS ssNMR experiments (Heise *et al.* 2005). Subsequently, Viennet and coworkers characterized the comprehensive interactions of α-Syn with various phospholipid nanodiscs in a quantitative and site-resolved manner, hypothesizing that the interaction between α-Syn and membranes may facilitate the primary nucleation step of amyloid fibrils (Viennet *et al.* 2018). Dhavale and coworkers amplified α-Syn fibrils derived from patients with DLB and constructed an atomic-resolution structure of α-Syn fibrils using ssNMR (Dhavale *et al.* 2024).

However, proteins are not merely simplified rigid objects, even stable and well-folded proteins possess flexible regions with varying degrees of mobility. Many disease-associated amyloid fibrils consist of an ordered rigid core and disordered terminal regions (Gallardo *et al.* 2020). The rigid regions confer stability, rigidity, and seeding capacity, while the intrinsically disordered region (IDR) complements the functions of the rigid regions and plays a significant role in pathological activities (Olzscha *et al.* 2011; Uversky 2013). Extensive structural knowledge of rigid fibril cores has been accumulated through the use of cryo-EM and CP-based ssNMR techniques (Li and Liu 2022; Tang *et al.* 2013), yet the IDR remains challenging to probe effectively due to their highly disordered nature. Consequently, there is a notable lack of understanding regarding the conformations of IDRs and their interactions with various binding proteins (receptors (Zhang *et al.* 2021), chaperones (Wentink *et al.* 2020), and proteasomes (Hong *et al.* 2014)) compared to the extensive research focusing on rigid regions.

The polarization transfer based on scalar coupling is insensitive to motion and can be effective in both rigid and flexible regions. Scalar coupling-based NMR experiments select signals with long transverse (T_2_) relaxation times, thereby prioritizing the detection of protein flexible regions. The T_2_ relaxation of the rigid regions is faster compared to that of the flexible regions; thus, during the transfer periods of the insensitive nuclei enhanced by polarization transfer (INEPT), the difference in T_2_ relaxation rates allows for the filtering out of signals from the rigid regions, leaving only those from the flexible regions (Loquet *et al.* 2009; Schanda and Ernst 2016; Tomaselli *et al.* 1998).

In previous studies, the application of ^1^H-^15^N two-dimensional (2D) heteronuclear single quantum coherence (HSQC) NMR for solid protein samples has enabled the acquisition of cross-peak signals from flexible region residues, yet it falls short in backbone assignment (Gopinath *et al.* 2017). In protein NMR, the separation of protein signals relies on correlations between multiple nuclei, and for complex systems such as membrane proteins or amyloid fibrils, an additional carbon dimension is necessary to achieve better dispersion of amide resonances. Recently, scalar coupling-based triple resonance ssNMR has been employed to characterize IDR in various proteins, including microcrystalline proteins, fibrils, membrane proteins, and large protein complexes (Andronesi *et al.* 2005; Falk and Siemer 2016; Gao *et al.* 2013; Linser *et al.* 2010, 2011). For instance, backbone assignment of the flexible regions of Tau and HET has been accomplished through scalar coupling-based ^1^H detection triple resonance ssNMR experiments (Bibow *et al.* 2011; Caulkins *et al.* 2018). Similarly, the flexible tails of histones in large protein complexes like nucleosomes, assembled from histone octamers and DNA, can be characterized using the same method (Xiang *et al.* 2018). Scalar coupling-based NMR experiments can also be utilized to characterize the rigid regions of extensively perdeuterated proteins. Linser and colleagues adapted a three-dimensional (3D) pulse sequence from solution NMR for application on solid-state microcrystalline samples. The H^N^ linewidth of the diluted perdeuterated (100% ^2^D on non-exchangeable sites and 10% ^1^H on exchangeable sites) samples is approximately 20 Hz, providing sufficiently long ^1^H T_2_ relaxation times for effective INEPT transfer, which can be used to complete the assignment of the solid protein backbone (Linser *et al.* 2008).

Given the significance of IDR in pathological activities, probing their high-resolution conformational information and interactions with ligands aids in further understanding the role of IDR in disease mechanisms. Scalar coupling-based ssNMR provides the essential technical conditions for IDR research. This protocol outlines the process of backbone assignment for amino acid residues of IDR in ^15^N/^13^C-labeled α-Syn fibrils using scalar coupling-based ^1^H detection MAS ssNMR. Furthermore, based on the backbone assignment of IDR, it facilitates site-specific monitoring of conformational changes in the IDR of α-Syn fibrils during receptor protein binding. This approach can be broadly applied to the study of IDR in other insoluble protein systems that exhibit detectable chemical shifts and sufficient homogeneity.

**OVERVIEW OF PROTOCOL**

**Figure 1:**
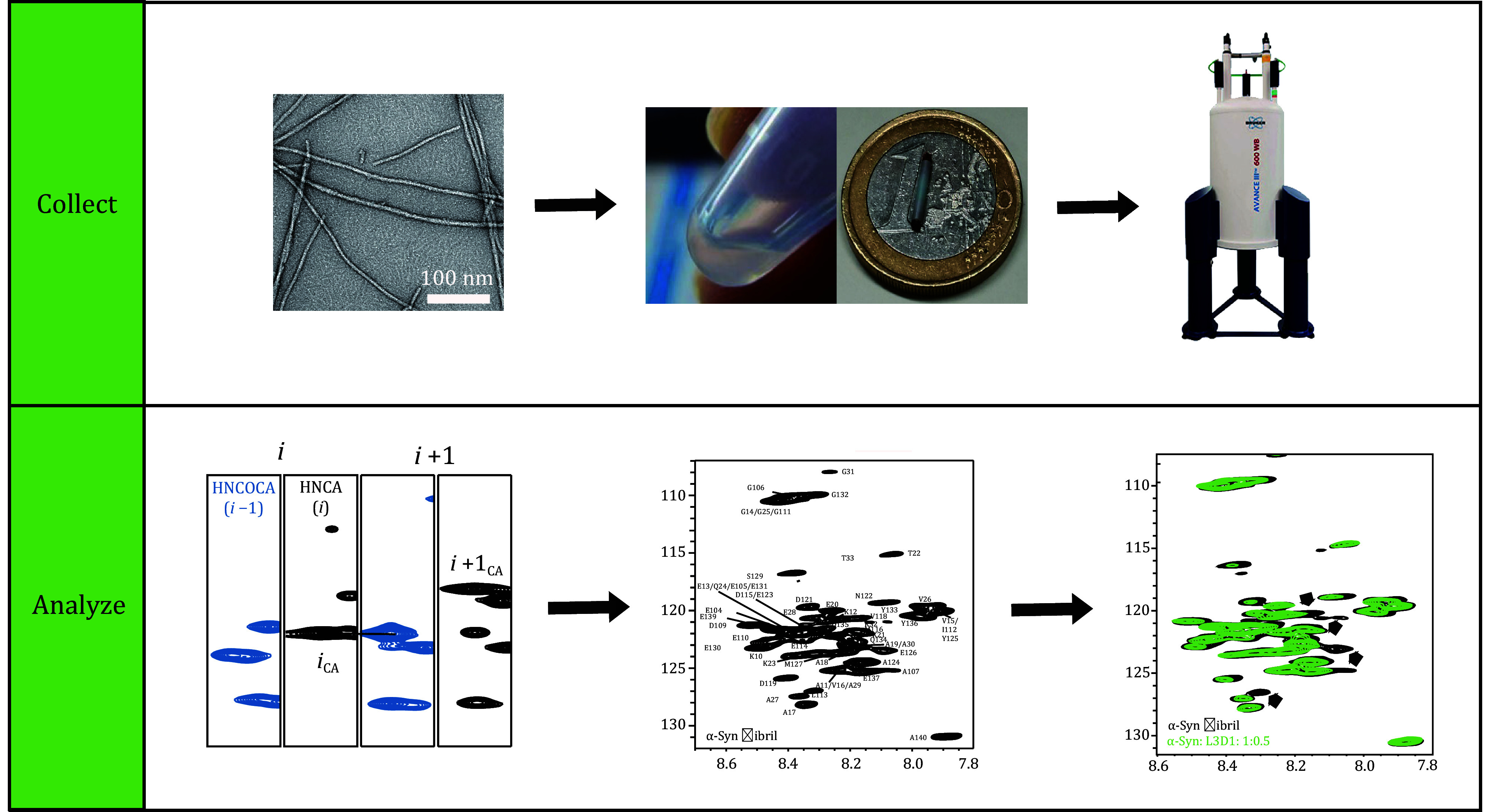
Technical scheme of the scalar coupling-based ^1^H detection MAS ssNMR. This is adapted with permission from Zhang *et al.* (2023)

The scalar coupling-based ^1^H detection MAS ssNMR method is a crucial technique for investigating the conformations and interactions within IDR. It offers advantages such as minimal sample requirements, reduced experimental time, and high sensitivity (Fricke *et al.* 2017). This study outlines the process of backbone assignment for uniformly ^15^N/^13^C-labeled α-Syn fibrils' IDR (Fig. 1).

Initially, α-Syn fibril samples with correct biophysical properties were obtained through ultracentrifugation and loaded into a 1.3-mm ssNMR rotor. 2D HSQC spectrum and 3D HNCO, HNCA, and HN(CO)CA spectra were collected on a 600-MHz ssNMR spectrometer. Subsequently, spectral data were analyzed to assign the backbone of the IDR.

In conclusion, this process represents an effective approach for characterizing IDR in amyloid fibrils and other insoluble protein complexes that may contain IDR. Ultimately, it is our hope that this method will further elucidate the conformational changes of IDR in the context of ligand binding processes.

## MATERIALS

### Preparation of ^15^N/^13^C-labeled α-Syn fibril

The expression and purification of ^15^N/^13^C-labeled α-Syn fibrils were conducted using previously established methods (Zhang *et al.* 2023). *Escherichia coli* containing the human α-Syn plasmid was cultured in M9 medium, utilizing ^15^NH_4_Cl as the sole nitrogen source and ^13^C_6_-glucose as the sole carbon source, with protein expression induced by isopropyl-β-D-1-thiogalactopyranoside (IPTG). The α-Syn was purified through ion exchange and size exclusion chromatography at 4°C. The α-Syn monomers were concentrated at 500 μmol/L and incubated at 37°C with agitation for one week to yield fibrils. Subsequently, the fibrillar samples were sonicated for 30 s to generate α-Syn fibril seeds. These seeds were then added to the ^15^N/^13^C-labeled α-Syn monomers and stirred at 900 r/min, incubating at 37°C for five days to obtain mature fibrils for ssNMR analysis. The mature fibrils should be kept at room temperature or frozen.

### Solid-state NMR spectroscopy

The ^15^N/^13^C-labeled α-Syn fibrils were subjected to ultracentrifugation at 150,000*g* for 120 min. The resulting gel-like pellet was placed into a 1.3-mm rotor. NMR experiments were performed at 60-kHz MAS on a wide-bore 600-MHz spectrometer equipped with a 1.3-mm HXY MAS probe. All NMR data were processed using TopSpin and analyzed with Sparky.

**[Tip]** The proton-detected experiments designed in this study can be conducted at 20-kHz MAS or less while maintaining the same temperature.

The main reagents used in the experiment are listed in Table 1. The main instruments and equipment used in the experiment are listed in Table 2.

**Table 1:** Reagents

Name	Manufacturer
Ammonium Chloride (^15^N, 99%)	Diamond
D-Glucose (U-^13^C6, 99%)	Diamond
Tris-HCl	Diamond
IPTG	Sangon Biotech
EDTA	Sangon Biotech
Phenylmethylsulfonylfluoride	Sangon Biotech
Streptomycin	Sangon Biotech
NaCl	Sinopharm Chemical Reagent Co., Ltd
KCl	Sinopharm Chemical Reagent Co., Ltd
Unstained Protein MW Marker	Thermo

**Table 2:** Instruments and equipment

Name	Model	Manufacturer
Mini Centrifuge	EQ-6K	Eastwin Scientific Equip-mentsinc
Centrifuge	Microfuge16	Beckman Coulter
Desktop refrigerated microcentrifuge	Microfuge20R	Beckman Coulter
Benchtop refrigerated centrifuge	Allegra X-15R	Beckman Coulter
High-Speed Refrigerated Centrifuge	AvantiJ-26SXP	Beckman Coulter
Benchtop Ultracentrifuge	Optima MAX-XP	Beckman Coulter
Fixed-Angle Rotor	TLA-55	Beckman Coulter
Analytical and Precision Balances	Practcum	Sartorius
Spectrophotometer	DS-11+	DeNovix
JN-Mini Pro Low-Temperature Ultra-high pressure cell disrupter	JN-Mini Pro	JNBIO
Ultrasonic Cell Disruptor	Scientz-IID	Ningbo Scientz Biotech-nology Co., Ltd
Vertical Electrophoresis System	Mini P-4	CAVOY
Gel Documentation and Analysis	GenoSens 2100	Clinx Science Instruments Co., Ltd.
SSNMR Rotor	1.3-mm	Bruker
600-MHz magnetic resonance spectrometer	Bruker Avance Neo WB 600-MHz	Bruker

## PROCEDURE

### Step 1: Spinning Stability Testing

After loading the sample into the rotor, ensure that the drive tip and the bottom cap are securely closed. Inspect the rotor for any deformation or scratches, and verify that both the drive tip and the bottom cap are intact. Place the rotor into the MAS testing platform for spinning stability testing. In the software interface, select the rotor type (and the probe type if necessary), entering parameters such as diameter (mm), minimum speed (Hz), and maximum speed (Hz). Gradually increase the speed to 20 kHz and maintain stability at this speed for 1–2 h before concluding the test.

**[Tip]** The rotor requires meticulous examination under a magnifying glass or dissecting microscope. The maximum spinning speed for stability testing is generally set at 20 kHz. If stable operation is achieved at this speed, it is highly likely that stability can also be maintained at higher speeds, such as 60-kHz. If the test needs to be performed at a higher speed, the cooling gas must be connected.

### Step 2: Spinning up

Insert the rotor into the 1.3-mm probe and gradually increase the spinning speed. During this process, monitor the bearing and drive pressures. For biological samples, measure the actual temperature of the sample by observing the water’s peak position to prevent freezing or overheating. It is generally accepted that at low speeds, the heat generated during rotation is minimal, and the sample temperature is approximately equal to the set temperature. As the spinning speed increases and frictional heat intensifies, the set temperature must be lowered, and the gas flow adjusted accordingly to ensure that the sample temperature does not become excessively high. Based on empirical data, the chemical shift of the water is approximately 4.96 ppm at 5°C, with the peak position decreasing by 0.011 ppm for every 1°C increase in temperature. When the desired spinning speed is reached, proceed to match and tune the ^1^H, ^13^C, and ^15^N channels.

**[Tip]** Temperature influences the state of the sample, thereby affecting the final signal. Since the biological samples always contain some water, the actual sample temperature can be monitored via the chemical shift of water. This method is not applicable to other anhydrous products. The actual temperature can be calculated using the following formula (applicable range: temperature 0–52°C, pH 2–7, salt concentration 0–1 mol/L) (Hoffmann *et al.* 2019). It is recommended to use 4,4-dimethyl-4-silapentane-1-sulfonic acid (DSS) as a chemical shift reference. It can also be assumed that at lower speeds, the actual temperature is equivalent to the set temperature for calibration purposes.

\begin{equation*}\begin{split}
T=\; & \text{0}\mathrm{{^\circ}}{\mathrm{C}} < T\leqslant \ \text{52} \mathrm{\mathrm{{^\circ}}{\mathrm{C}}},\ \text{470.7} \mathrm{{^\circ}}{\mathrm{C}}-\text{93.8}\frac{\mathrm{{^\circ}}{\mathrm{C}}}{\text{ppm}} \\ &\times \Bigg[\delta_{\text{H}_{\text{2}}\text{O}}-\text{0.002}\frac{\text{ppm}}{\text{pH unit}}\times \left(\text{pH}-\text{7.4}\right) \\ &-\text{9}\times \text{10}^{-5}\frac{\text{ppm}}{\text{m}\text{mol/L}}\times \text{C}_{\text{salt}}\Bigg]\ .
\end{split}\end{equation*}

### Step 3: Calibration and setting of pulse parameters (Table 3)

**Table 3:** The pulse parameters for acquiring scalar coupling-based spectra at a 600-MHz spectrometer at 60-kHz MAS

Parameter	Value
90° initial ^1^H excitation pulse	
Duration (µs)	1.35
Amplitudes (kHz)	185
Power level (w)	35
90° ^15^N flip pulses	
Duration (µs)	4.5
Amplitudes (kHz)	55.5
Power level (w)	30
^1^H decoupling pulse	
Duration (µs)	80
Amplitudes (kHz)	3.125
Power level (w)	0.009977
^15^N decoupling pulse	
Duration (µs)	100
Amplitudes (kHz)	2.5
Power level (w)	0.061
^13^C soft pulse	
90° Q5 pulse duration (µs)	400
180° Q3 pulse duration (µs)	256

Step 3.1: Calibration of the 90° pulses for ^1^H, ^15^N, and ^13^C is performed using a CP-based standard calibration method. Initially, input the routine values for pulse length and power levels to obtain a preliminary view of the CP spectrum signal.

^1^H: Double the length of the first 90° pulse for ^1^H and adjust its length until the signal completely disappears. At this point, the pulse length corresponds to a 180° pulse length at the given power. Half of this value will be the 90° pulse length.

^15^N: After the contact pulses of the ^1^H-^15^N CP, introduce a hard pulse on ^15^N, positioning the carrier at the center of the ^15^N signal region. Adjust its length until the signal disappears completely. The pulse length at this stage corresponds to a 90° pulse length at the specified power.

^13^C: The calibration method for ^13^C is similar to that of ^15^N. The carrier position should first be set to the center of the ^13^CO region. Due to the narrow distribution of the ^13^CO signal and its non-overlapping nature with other signals, it allows for better observation of the zero point. The remaining procedures are the same as those used in the ^15^N calibration.

Step 3.2: Set the duration of the initial 90° proton excitation pulse to 1.35 µs and the duration of the ^15^N 90° flip pulse to 4.5 µs, and calculate and set their corresponding power levels, respectively. Set the ^13^C 90° soft pulse duration to 400 µs and the 180° soft pulse duration to 256 µs, and calculate the corresponding power level based on the 90° hard pulse in the “shape tool” within TopSpin.

Step 3.3: During the ^1^H signal acquisition process, 2.5-kHz radio frequency (RF) field is applied on the ^15^N channel for ^1^H-^15^N WALTZ-16 decoupling. During the free evolution of ^15^N and ^13^C, a 3.125-kHz RF field is applied for ^1^H DIPSI-2 broadband decoupling. The proton channel utilizes a high-performance MISSISSIPPI water suppression module. The duration of a single water-suppression pulse is approximately 10 ms, with a power level ranging from 0.1 to 0.5 w (Zhou and Rienstra 2008).

**[Tip]** The amplitudes of the decoupling pulse must significantly exceed that of the scalar coupling. Once the power level of the decoupling pulse is determined, the corresponding duration can be calculated based on the duration and power level of the 90° hard pulse.

\begin{equation*}\begin{split}
 & \left(\frac{\text{90}{^\circ} \text{ pulse amplitudes}\; \text{(kHz)}}{\text{decoupling amplitudes}\; \text{(kHz)}}\right)^{\text{2}} \\ &=\frac{\text{90}{^\circ} \text{ pulse power level}\; \text{(w)}}{\text{decoupling power level}\; (\text{w})}\ ,
\end{split}\end{equation*}

\begin{equation*}\begin{split} &
 \text{90}{^\circ}\text{ pulse amplitudes}\;\text{(kHz)} \\&
 = \frac{\text{1}}{\text{4}\times\text{90}{^\circ} \text{ pulse duration}\;(\mu\text{s})} .
\end{split}\end{equation*}

**[? TROUBLESHOOTING]**

### Step 4: Set the parameters for the 2D ^1^H-^15^N refocused-HSQC experiment

The parameters are outlined in Tables 3 and 4.

**Table 4:** The experimental parameters for acquiring multidimensional spectra

	^1^H	^15^N	^13^C
2D HSQC			
Acquisition time (ms)	40	40	
Spectral width (ppm)	19.8	28	
Offset (ppm)	4.728	120	
Real points	1024	170	
3D HNCO			
Acquisition time (ms)	40	19	9.5
Spectral width (ppm)	19.8	26	7
Offset (ppm)	4.728	119	173.5
Real points	952	60	20
3D HNCA&HN(CO)CA			
Acquisition time (ms)	40	14.5	6.9
Spectral width (ppm)	19.8	26	23
Offset (ppm)	4.728	119	51.7
Real points	952	46	48

Step 4.1: Set the acquisition time in the ^1^H direct dimension to 40 ms and that in the ^15^N indirect dimension to 40 ms.

**[Tip]** The maximum acquisition time for the indirect dimension is contingent upon the characteristics of the sample. Exploratory pre-experiments can be conducted by initially setting the acquisition time to 20–30 ms, followed by adjustments based on the signal decay observed.

**[? TROUBLESHOOTING]**

Step 4.2: In the direct dimension (^1^H), set the spectral width to cover the entire proton spectrum (19.8 ppm), set the RF offset to 4.728 ppm to align with the water peak position, and set the number of real points to 1024, corresponding to the aforementioned 40 ms acquisition time. In the indirect dimension (^15^N), set the spectral width to cover the entire amide nitrogen range (28 ppm), set the RF offset to 120 ppm, and set the number of real points to 170, also corresponding to the aforementioned 40 ms acquisition time.

Step 4.3: Set the number of scans to 32 and the number of dummy scans to 8. Set the recycle delay to 2 s.

**[Tip]** The number of scans is typically set as an integer multiple of the phase cycling steps. The optimal recycle delay time varies among different samples and should be determined experimentally.

Step 4.4: Implement the uniform sampling scheme with the States-TPPI method in the indirect dimension. Within the data processing window, configure the zero-filling amount and apply a squared cosine window function with phase shifting to process the spectra across all dimensions.

Step 4.5: Input “zg” to initiate the recording of the 2D ^1^H-^15^N refocused-HSQC spectrum.

**[? TROUBLESHOOTING]**

### Step 5: Set the parameters for the 3D HNCO, HNCA, and HN(CO)CA experiments

The parameters are outlined in Tables 3 and 4.

Step 5.1: For the HNCO experiment, set the acquisition times to 40 ms for the^ 1^H direct dimension, 19 ms for the ^15^N indirect dimension, and 9.5 ms for the ^13^C indirect dimension.

For the HNCA and HN(CO)CA experiments, set the acquisition times to 40 ms for the ^1^H direct dimensions, 14.5 ms for the ^15^N indirect dimensions, and 6.9 ms for the ^13^C indirect dimensions.

Step 5.2: The spectral widths and RF offsets for ^1^H direct dimension and ^15^N indirect dimension can be referenced from the parameters of 2D NMR experiments (Step 4.2). For the HNCO experiment, set the spectral width to span the whole ^13^CO region in the ^13^C indirect dimension and set the RF offset to the center of the ^13^CO region; for the HNCA and HN(CO)CA experiments, set the spectral widths to span the whole ^13^CA region in the ^13^C indirect dimension and set the RF offsets to the center of ^13^CA region. A list of optimized parameters can be found in Table 4.

**[Tip]** The spectral widths required for the indirect dimensions and the duration of the signals can be derived from the 2D ssNMR experiments. To effectively utilize the measurement time, set the spectral widths of the indirect dimensions as accurately as possible and optimize the necessary data points for the indirect dimensions. Due to differing reference standards, the carbon chemical shift values between TopSpin and Biological Magnetic Resonance Bank (BMRB) vary by approximately 2.8 ppm.

Step 5.3: For the HNCO and HNCA experiments, set the number of scans to 224 and the number of dummy scans to 8. Set the recycle delay to 2 s.

For the HN(CO)CA experiment, set the number of scans to 320 and the number of dummy scans to 8. Set the recycle delay to 2 s.

Step 5.4: Employ the Non-Uniform Sampling (NUS) scheme with the States-TPPI method in the indirect dimension. The sampling ratio for the HNCO experiment is 50%, resulting in the acquisition of 150 complex points (equal to 600 real points). The sampling ratio for HNCA and HN(CO)CA experiments is 25%, resulting in the acquisition of 138 complex points (equal to 552 real points).

**[Tip]** NUS scheme reduces sampling time. Through the application of relevant algorithms and software, such as SMILE (Ying *et al.* 2016), istHMS (Hyberts *et al.* 2012), and MDD (Jaravine *et al.* 2006), missing data points can be reconstructed.

Actual number of complex points = Planned complex points of the N dimension × Planned complex points of the C dimension × sampling ratio.

Step 5.5: Input “zg” to initiate the recording of the 3D HNCO, HNCA, and HN(CO)CA spectra.

**[? TROUBLESHOOTING]**

**[TIMING]**

The overall measurement time of the experiment is influenced by various factors, including the instrumentation, the rotor diameter, the sample conditions and the pulse sequence parameters. Table 5 illustrates the time necessary to record ssNMR spectra of uniformly ^15^N/^13^C-labeled α-Syn fibrils in a 1.3-mm rotor at a 600-MHz spectrometer at 60-kHz MAS.

**Table 5:** Measurement time

Experiment	Measurement time
HSQC	3 h, 33 min, 59 s
HNCO	3 d, 14 h, 31 min, 38 s
HNCA	3 d, 9 h, 44 min, 9 s
HN(CO)CA	5 d, 16 h, 27 min, 20 s

**[? TROUBLESHOOTING]**

Troubleshooting advice is shown in Table 6.

**Table 6:** Troubleshooting table

Step	Problem	Possible reason	Solution
3.3	Water peak suppression is not ideal	The parameters of the water peak suppression module are inappropriate	Systematically scan the parameters of the water peak suppression module
4.1	The overall measurement time is too long	(1) The acquisition time in each dimension is too long. (2) The number of real points is excessive	(1) Optimize the acquisition time according to the signal decay in the FID (2) Optimize the number of real points; Employ the non-uniform sampling scheme
4.5, 5.5	Signal intensity is low	(1) The sample amount is insufficient (2) The overall measurement time is insufficient	(1) Increase the sample volume loaded in the rotor. (2) Adjust overall measurement time according to the signal to noise ratio
4.5, 5.5	The spectral peak width is broad	Sample heterogeneity	Optimization of sample status
4.5, 5.5	The number of spectral peaks is excessive	(1) The number of amino acids is excessive (2) The presence of heterogeneous polymers in the sample	(1) Conduct higher-dimensional NMR experiments; Use protein segmentation labeling method to reduce peak number (2) Optimize the protein purification process

## ANTICIPATED RESULTS

**Figure 2:**
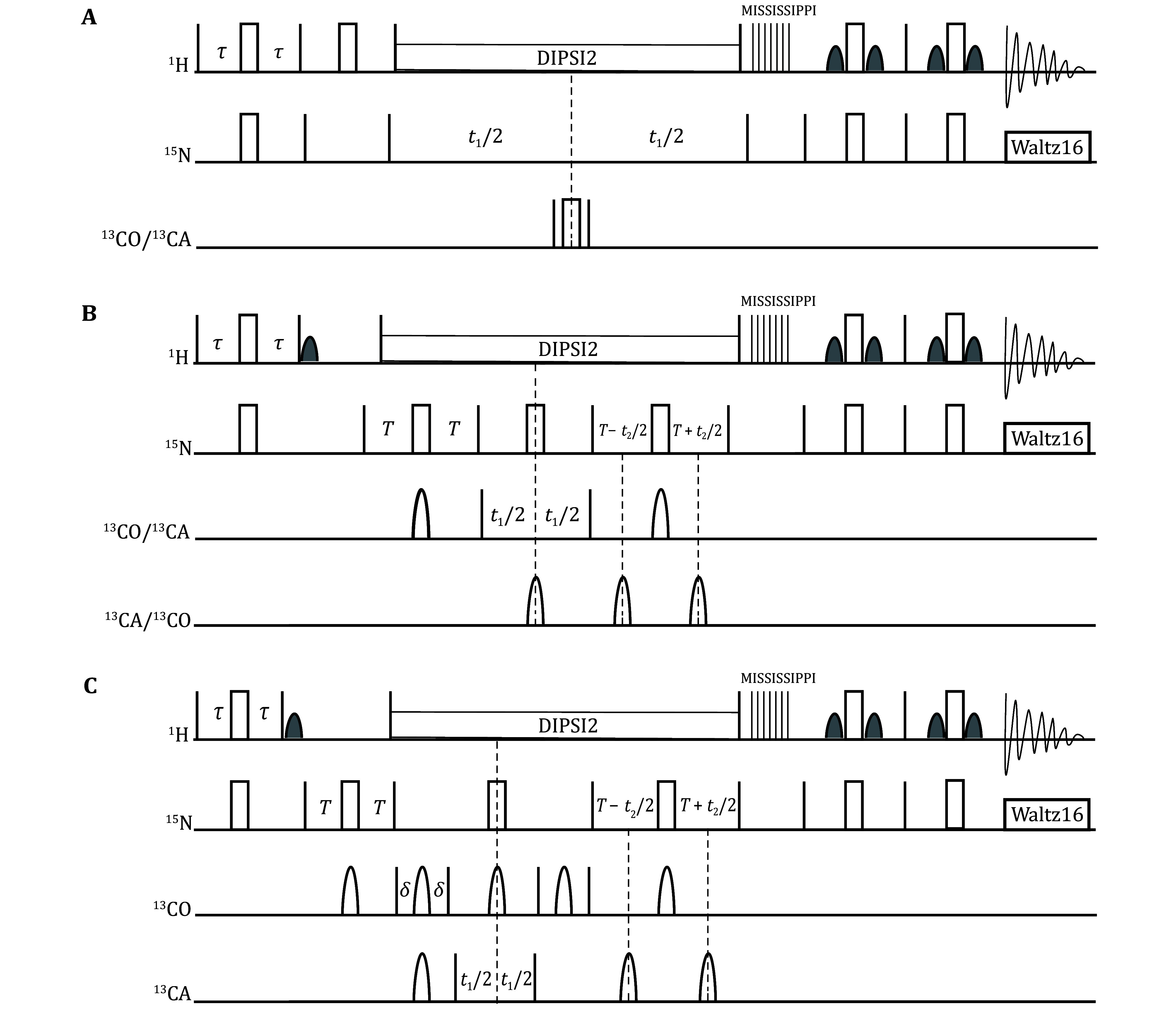
**A** Schematic diagram of the 2D ^1^H-^15^N refocused-HSQC experiment pulse sequence. The filled and empty rectangles represent 90° and 180° pulses, respectively. In the ^13^C channel, a 90°*x*-180°*y*-90°*x* composite pulse is applied at 113 ppm to, simultaneously decouple N-CA and N-CO. **B** Schematic diagram of the 3D HNCO and HNCA experiment pulse sequences, with *τ* and *T* set to 2.3 ms and 12.0 ms, respectively. Apart from the ^13^C carrier frequency, both pulse sequences are identical. The 90° pulse in the carbon channel is a Q5-shaped pulse of 400 µs duration, while the 180° pulse is a Q3-shaped pulse of 256 µs duration. **C** Schematic diagram of the 3D HN(CO)CA experiment pulse sequence, with *τ*, *T* and *δ* set to 2.3 ms,12.0 ms and 4 ms, respectively. The 90° pulse in the carbon channel is a Q5-shaped pulse of 400 µs duration, while the 180° pulse is a Q3-shaped pulse of 256 µs duration

**Figure 3:**
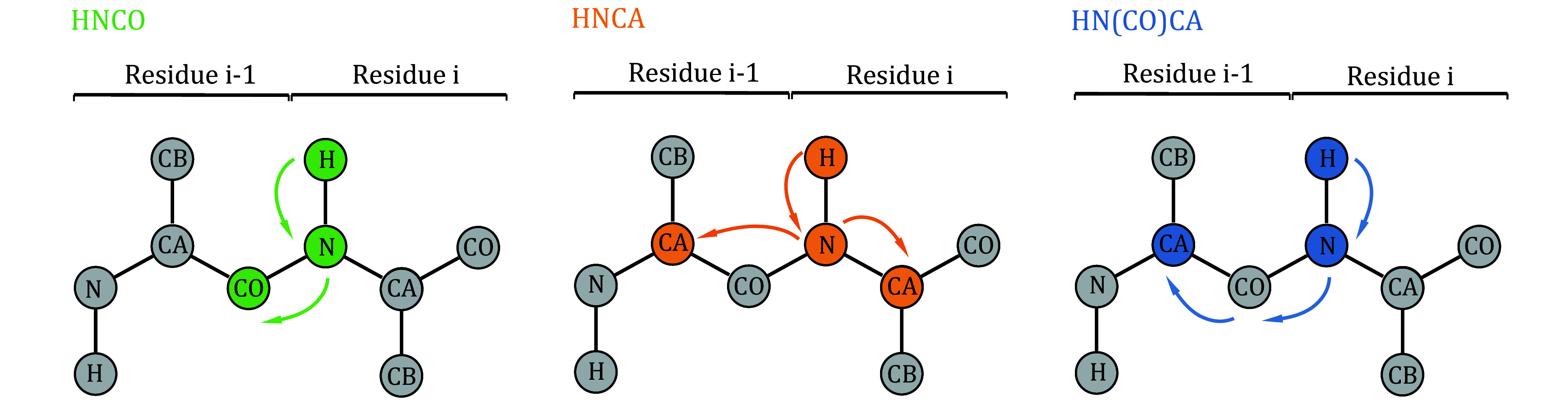
Schematic diagram of the spin polarization transfer pathways in the 3D HNCO, HNCA, and HN(CO)CA experiments, highlighting two adjacent residues (residues i-1 and i) within the protein backbone. Arrows indicate the polarization transfer process. Colored circles represent the nuclei for which chemical shifts were correlated during the experiment

**Figure 4:**
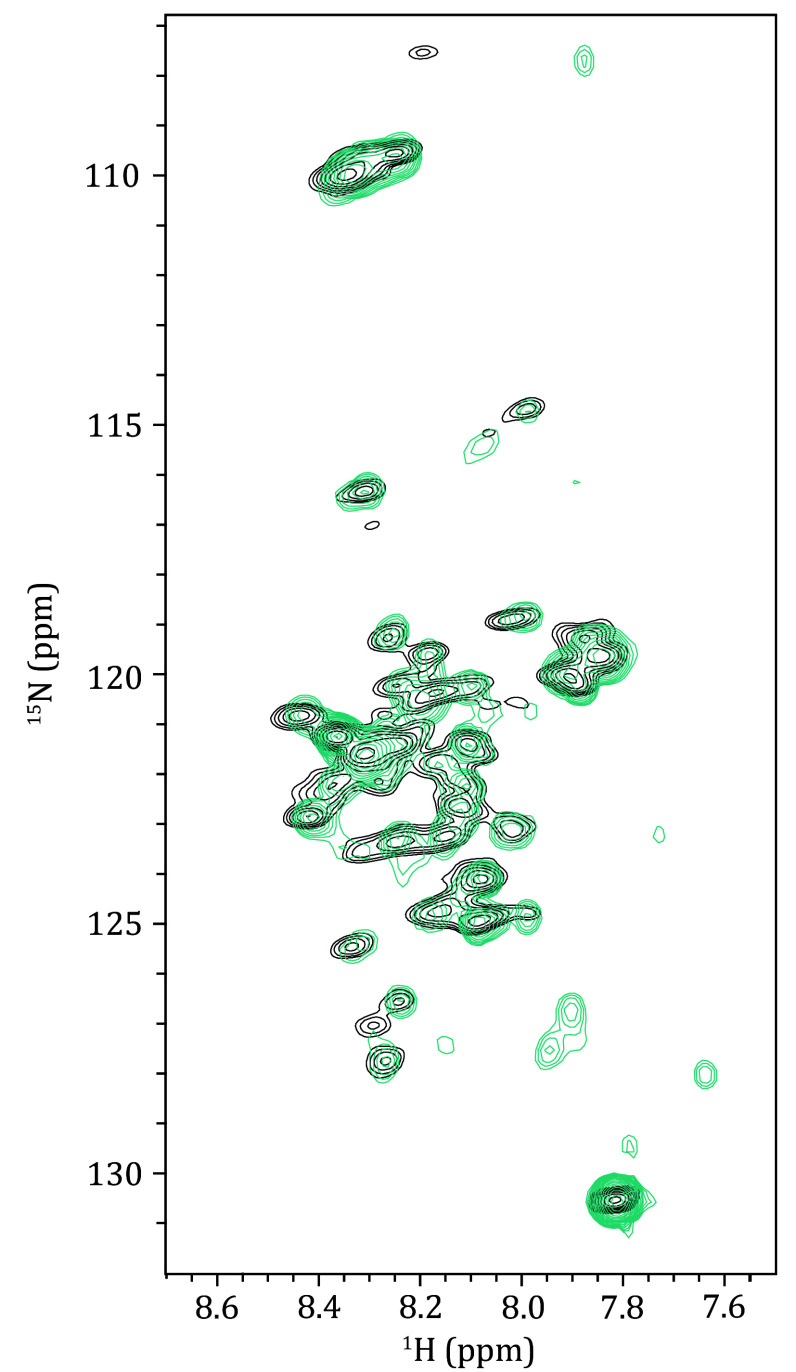
Overlay of the 2D ^1^H-^15^N projection of the 3D HN(CO)CA spectrum (turquoise) onto the 2D ^1^H-^15^N refocused-HSQC spectrum (black). The non-overlapping peaks at the lower right may be due to sample degradation during the experimental process

**Figure 5:**
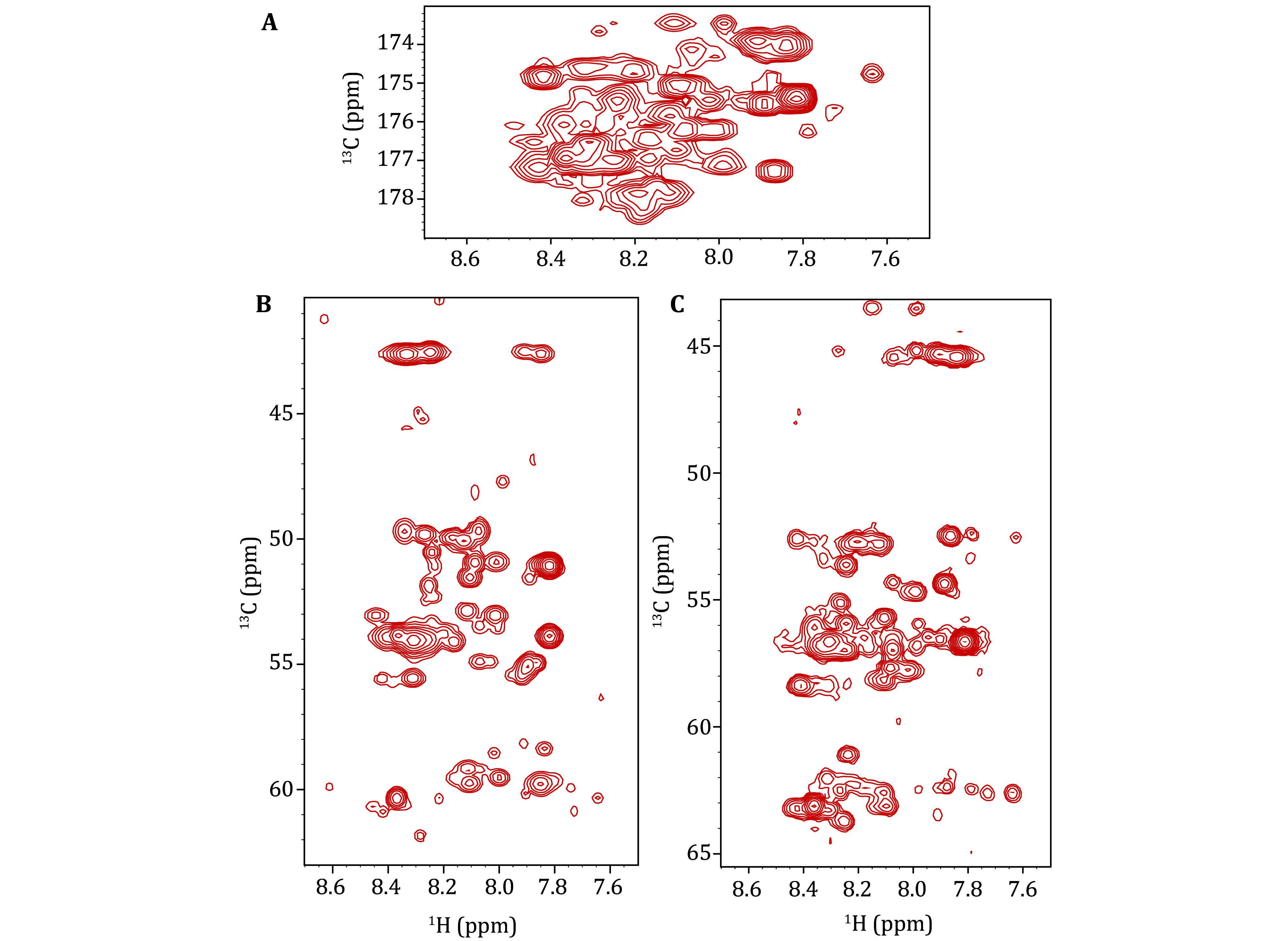
**A** 2D ^1^H-^13^CO projection of the 3D HNCO spectrum of uniformly ^15^N/^13^C-labeled α-Syn fibrils recorded in a 1.3-mm rotor at a 600-MHz spectrometer at 60-kHz MAS, illustrating the signal pattern of the sample. **B** 2D ^1^H-^13^CA projection of the 3D HNCA spectrum. **C** 2D ^1^H-^13^CA projection of the 3D HN(CO)CA spectrum. It is noteworthy that the signals appearing in the ^1^H dimension at 7.8–7.6 ppm are attributed to sample degradation

The pulse sequences are illustrated in Fig. 2, while the spin polarization transfer pathways are depicted in Fig. 3. By executing the aforementioned steps, a 2D NMR spectrum (Fig. 4) and three 3D NMR spectra (Fig. 5) can be obtained. The ssNMR pulse sequences correlate the nuclei of residue i in the protein sequence with those of the adjacent residue i-1.

**Figure 6:**
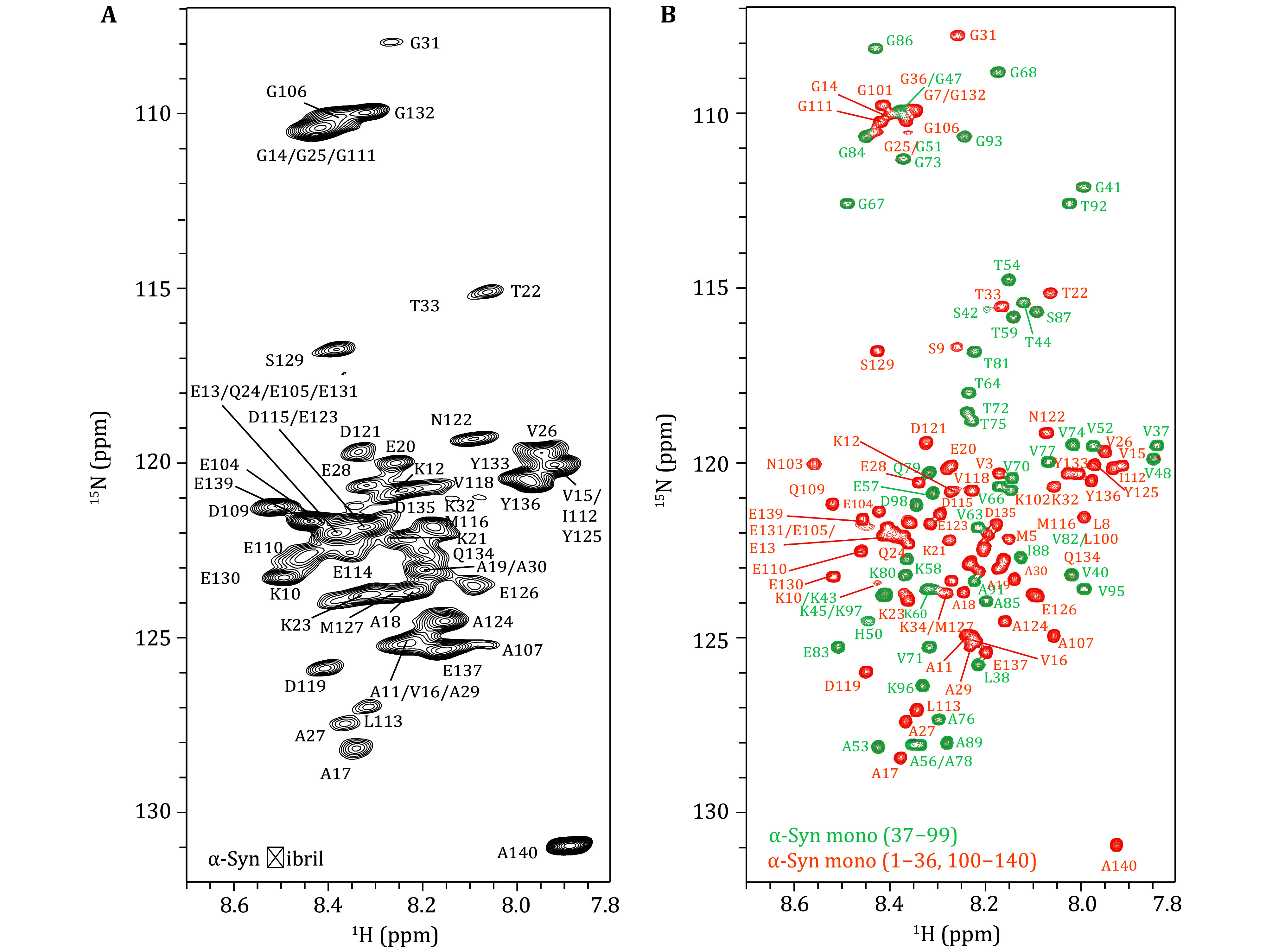
**A** 2D ^1^H-^15^N refocused-HSQC spectrum of uniformly ^15^N/^13^C-labeled α-Syn fibrils recorded in a 1.3-mm rotor at a 600-MHz spectrometer at 60-kHz MAS, with assigned residues marked. The clustering of residue signals in the 7.8 to 8.6 ppm range indicates the detection of the IDR of α-Syn fibrils. **B** 2D ^1^H-^15^N HSQC spectrum of uniformly ^15^N/^13^C-labeled α-Syn monomers. This is adapted with permission from Zhang *et al.* (2023)

In the 2D ^1^H-^15^N refocused-HSQC ssNMR experiment, polarization is transferred from the hydrogen nuclei to the amide nitrogen nuclei, resulting in the formation of cross peaks for each residue, which constitute the fingerprint spectrum of the IDR of α-Syn fibrils (Fig. 6A). The pattern is comparable to the 2D ^1^H-^15^N HSQC solution NMR spectrum of α-Syn monomers (Fig. 6B), as the IDR retains a similar disordered state within the solid fibrils as observed in the liquid state. This similarity allows for the transfer of assignments from the existing liquid-state data to the solid-state spectra. Select characteristic peaks to align the two spectra. Since some signals are located at the edges of the spectrum where signals are sparse, such assignments can already be obtained through the direct transfer on the 2D spectrum. However, for the majority of signals, direct transfer on the 2D spectrum is not feasible due to peak overlap, necessitating the assistance of 3D NMR spectra.

The inclusion of chemical shifts for CA and CO in the 3D NMR spectra enhances resolution. Additionally, characteristic CA or CO chemical shift values aid in identifying residue types, thereby facilitating better mapping to the sequence.

**Figure 7:**
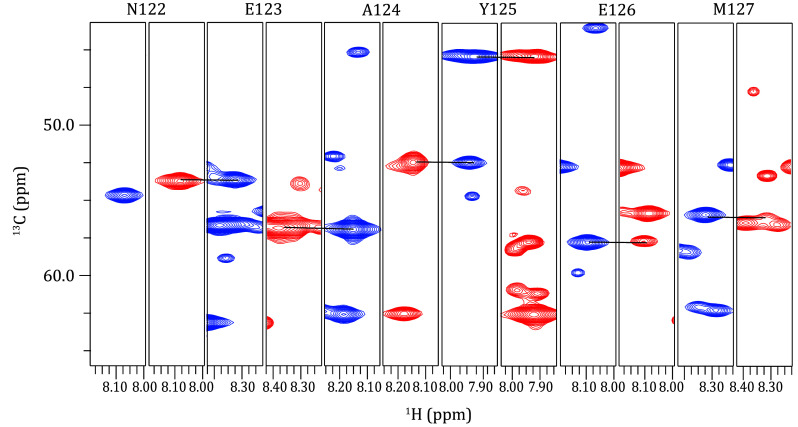
Sequential backbone walks from N122 to M127 of the ^15^N/^13^C-labeled α-Syn fibrils, achieved through paired HN(CO)CA (left, blue) and HNCA (right, red) experiments. Each strip is 0.2 ppm wide. The HNCA and HN(CO)CA spectra can complete the assignment of most signals in the HSQC spectrum of α-Syn fibrils. This is adapted with permission from Zhang *et al.* (2023)

A chemical shift comparison is performed between the CO and CA signals in the 3D HNCO and HNCA ssNMR spectra and the corresponding regions in the 3D solution NMR spectra. This simultaneous utilization of chemical shift similarities across three dimensions allows for the transfer of amino acid assignments. Furthermore, the 3D HNCA and HN(CO)CA ssNMR spectra provide information on the sequential relationships of backbone residues, enabling cross-validation of the transfer results. The HNCA experiment yields signals for H_i_^N^-N_i_^H^-C^α^_i_ and H_i_^N^-N_i_^H^-C^α^_i-1_ (Fig. 3), while the HN(CO)CA experiment provides the signal for H_i_^N^-N_i_^H^-C^α^_i-1_ (Fig. 3). By integrating these two experiments, a direct sequential connection can be established through the match of CA chemical shifts (Fig. 7).

Through this process, the backbone assignment of the IDR of α-Syn fibrils is progressively completed and annotated on the 2D ^1^H-^15^N refocused-HSQC spectrum (Fig. 6A).

**Figure 8:**
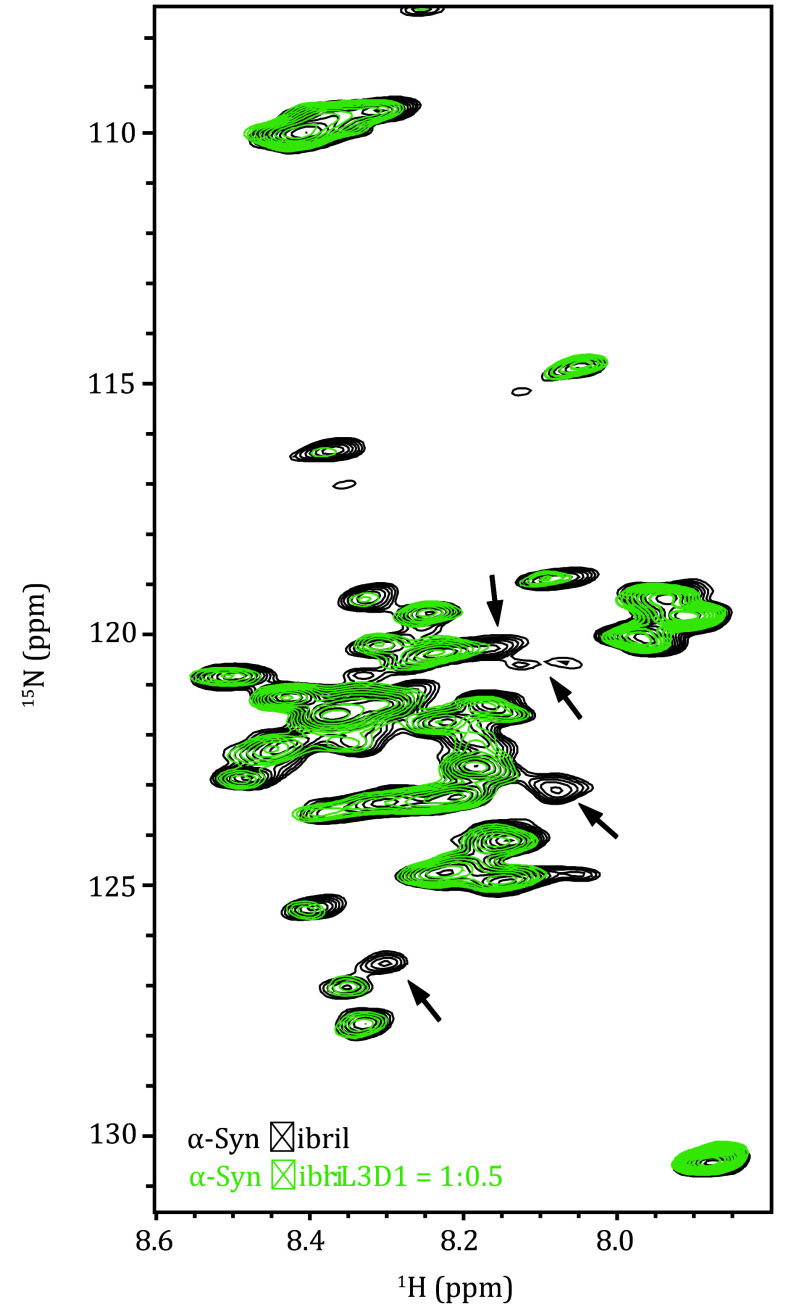
Overlay of the 2D ^1^H-^15^N refocused-HSQC spectra of ^15^N/^13^C-labeled α-Syn fibrils in the absence (black) and presence of L3D1 (green), with an α-Syn to L3D1 molar ratio of 1:0.5. Arrows indicate several prominent spectral peaks that have disappeared. This is adapted with permission from Zhang *et al.* (2023)

Following the backbone assignment, the scalar coupling-based ^1^H detection MAS ssNMR method can provide high-resolution interaction information. For α-Syn fibrils, we collected a 2D ^1^H-^15^N refocused-HSQC spectrum (Fig. 8) at a binding ratio of 1:0.5 between α-Syn fibrils and the L3D1 receptor. By comparing this with the spectrum of free α-Syn fibril, we can monitor the conformational change with a residual level resolution. We aim to further elucidate the conformational changes of the IDR of amyloid fibrils during the binding process with different ligand proteins, thereby deepening our understanding of the significant role that amyloid fibrils play in the pathogenesis of related diseases.

## Conflict of interest

Tong Zeng, Juan Li, Chaowei Shi and Shengqi Xiang declare that they have no conflict of interest.
